# 

**Published:** 1908-08-01

**Authors:** 


### CONTENTS.

| | PAGE |
|---|---|
| **The Teaching of Stoicism** | 459 |
| **The British Medical Association** | 460 |
| **Annotations—** | |
| Tobacco Smoking—The Poisons and Pharmacy Bill—The Storage of River Water | 461 |
| **Medical Opinion and Movement** | 462 |
| **Hospital Clinics—** | |
| The Treatment of Acute Pneumonia. By J. Walter Carr, M.D., F.R.C.P. (Lond.) | 465 |
| Prof. P. F. Richter on some New Ideas on Diseases of Metabolism | 467 |
| **Medicine—** | |
| Enlargements of the Spleen—II. | 469 |
| Cystitis due to Bacillus Coli Communis | 470 |
| **Surgery—** | |
| Œsophageal Obstruction.—I. | 471 |
| Unsuspected Fractures | 472 |
| **Obstetrics—** | |
| Inevitable Abortion and its Treatment | 473 |
| **Pathology—** | |
| The Alkalinity of the Blood in Disease | 474 |
| **Anæsthetics—** | |
| The Administration of Anæsthetics to Children | 475 |
| **Therapeutics—** | |
| The Preparation of Hot Compressed Air | 476 |
| **Laryngology and Rhinology—** | |
| Nasal Obstruction—III. | 477 |
| **Ophthalmology—** | |
| Cyclitis and Irido-Cyclitis—II. | 478 |
| **Life Assurance—** | |
| Practical Hints upon Medical Examination for Life Assurance—I. | 479 |
| **The General Practitioner's Column—** | |
| A Disease of the Skin occurring on the Hands of Workers among Sheep | 480 |
| **Hospital Administration—** | |
| Some Modern Continental Hospitals | 481 |
| **Nursing Administration—** | |
| Probationers in Excess—II. | 483 |
| **News and Coming Events** | 484 |
| **New Appliances and Things Medical** | 484 |

